# Optimal estimation of entanglement and discord in two-qubit states

**DOI:** 10.1038/s41598-019-39334-8

**Published:** 2019-02-28

**Authors:** Salvatore Virzì, Enrico Rebufello, Alessio Avella, Fabrizio Piacentini, Marco Gramegna, Ivano Ruo Berchera, Ivo Pietro Degiovanni, Marco Genovese

**Affiliations:** 1 INRIM, Strada delle Cacce 91, 10135 Torino, Italy; 2 Università degli Studi di Torino, Dipartimento di Fisica, Via Giuria 1, 10125 Torino, Italy; 3 Politecnico di Torino, Corso Duca degli Abruzzi 24, I-10129 Torino, Italy

## Abstract

Recently, the fast development of quantum technologies led to the need for tools allowing the characterization of quantum resources. In particular, the ability to estimate non-classical aspects, e.g. entanglement and quantum discord, in two-qubit systems, is relevant to optimise the performance of quantum information processes. Here we present an experiment in which the amount of entanglement and discord are measured exploiting different estimators. Among them, some will prove to be optimal, i.e., able to reach the ultimate precision bound allowed by quantum mechanics. These estimation techniques have been tested with a specific family of states ranging from nearly pure Bell states to completely mixed states. This work represents a significant step towards the development of reliable metrological tools for quantum technologies.

## Introduction

The problem to quantifying the amount of quantum resources in physical systems is strongly acknowledged by the physicists community, both for applications concerning quantum information technologies and experiments on quantum mechanics foundations.

The reconstruction of the density matrix, by means of the quantum state tomography, provides all the information on the physical system under analysis^1,2^. However, quantum state tomography is a demanding procedure in terms of quantum resources due the high number of measurements required on identical copies of the system. Moreover, it has two main limitations that could be critical for several applications: First of all, reconstructions are based on optimisation algorithms applied to likelihood functions, therefore, a tomography does not allow to perform an easy estimation of the uncertainty associated to the reconstructed density matrix. On the other hand, quantum state tomography becomes impractical for high-dimensional systems^3,4^. In addition, a full knowledge of the density matrix does not provide an immediate quantification of the amount of the quantum resource needed, hence, it is necessary introduce dedicated parameters.

Among the most relevant and exploited quantum resources, a crucial role is played by entanglement and discord, whose estimation is of the utmost relevance for present and upcoming quantum technologies. In general, the parameters used to evaluate them are defined for well specific families of quantum states, and several measurements have to be performed in order to experimentally obtain their values.

In particular, the measurement of the amount of entanglement is a parameter estimation problem where the value of entanglement is obtained indirectly from the measurement of one or more proper observables. A quantitative measure of entanglement corresponds to a non-linear function of the density operator, and it is not possible to identify a quantum observable directly associated to it. Several theoretical and experimental works have addressed this topic^5–8^, providing different approaches to efficiently estimate the amount of entanglement of a quantum state from a reduced set of measurements^9–13^, e.g. visibility measurements^14^, Bell tests^15^, entanglement witnesses^16–20^, Schmidt number^21–23^. Many of these techniques have also been implemented in laboratory^24–32^.

Quantum discord, instead, is a figure of merit that can be used to quantify non-classicality of correlations within a physical system^33–39^. Separability of the density matrix describing a multi-partite state does not guarantee vanishing of the discord, demonstrating that absence of entanglement does not imply classicality. Quantum discord has been proposed as the key resource needed for certain quantum communication tasks and quantum computational models not entirely relying on entanglement. Due to the high interest on quantum discord, both for foundational aspects of quantum mechanics and for applications, techniques allowing to estimate this quantity are demanded. Unfortunately, in general, quantum discord doesn’t present an analytical expression. Therefore, we take in account a geometrical approximation^40^ for our extimation task.

In many applications, specially for the quantum information technologies, a robust and resource-efficient protocol to estimate such quantities is highly demanded. Therefore, the optimisation problems concerning the ultimate precision bounds on entanglement and the optimal measurements achieving those bounds have been investigated^41,42^. That procedure is self-consistent and allows reaching the ultimate precision imposed by the quantum Cramér-Rao bound^43^, i.e. the minimum theoretical uncertainty compatible with the local quantum estimation theory^44–48^, obtained by maximizing the Fisher Information^43,49^.

Here, we exploit three different parameters^50^ providing quantitative information on the amount of entanglement in qubit states: Negativity, Log-Negativity and Concurrence. For each of these parameters, we introduce two different estimators: one non-optimal and one optimal (i.e. saturating the quantum Cramér-Rao bound). In addition to entanglement, we also introduce an optimal procedure to estimate Quantum Geometric Discord^51,52^, providing the best analytical approximation of the amount of quantum discord for the family of states under test (defined below). Actually, optimal estimators, when this simple, are an excellent solution in practical applications. However, we have introduced both optimal and non-optimal estimators for each parameter, in order to provide a direct comparison between the uncertainties in these two cases to highlight the advantage granted by optimal estimators.

This effort represents a sharp advancement with respect to our previous work^41,42^, since here we extend the entanglement estimation analysis to different parameters (Log-Negativity and Concurrence) and we address for the first time optimal estimation of quantum discord.

The paper is organised as follows: first of all, we introduce the estimators and related precision bounds obtained according to quantum estimation theory. Then, we describe our experiment aiming to estimate the amount of entanglement and discord of a large class of two-photon states. Finally, we compare experimental results, and their related uncertainty, with the theoretically-expected ones.

## Estimators Definition

We consider four different parameters: Negativity, Log-Negativity, Concurrency and Quantum Geometric Discord, allowing to quantify the amount of entanglement or discord in two-qubit systems. For each parameter, we introduce two estimators, one optimal and one non-optimal, allowing to estimate it with a smaller number of measurements with respect to a full reconstruction of the density matrix. However, to define such estimators, we need some *a priori* knowledge of the family of quantum systems we are going to test. In particular, our estimators are suited for quantum states whose density matrix can be expressed in the following form:

1 $$\rho =(1-p)(\begin{array}{cccc}0 & 0 & 0 & 0\\ 0 & q & 0 & 0\\ 0 & 0 & 1-q & 0\\ 0 & 0 & 0 & 0\end{array})+p(\begin{array}{cccc}0 & 0 & 0 & 0\\ 0 & q & -\,\sqrt{q(1-q)} & 0\\ 0 & -\,\sqrt{q(1-q)} & 1-q & 0\\ 0 & 0 & 0 & 0\end{array})$$

where *p* and *q* are unknown variables within the interval [0, 1]. This includes states with different entanglement amount, ranging from the singlet state (maximally entangled) to a completely de-coherent mixture. These are typical quantum states involved in many real scenarios in which entangled qubits are exposed to decoherence due to coupling with the environment, degradating the quantum resources available for the task we want to use them for. This makes them particularly worth investigating.

In the following, for each parameter, we define the estimators and we calculate the corresponding theoretical minimal uncertainty.

## Negativity

Negativity of entanglement is defined by:

2 $${\mathscr{N}}=\Vert {\rho }^{{T}_{A}}\Vert -1,$$

where: ${\rho }^{{T}_{A}}$ is the partial transpose of *ρ* with respect to the subsystem *A* and $\Vert X\Vert =Tr\,\sqrt{{X}^{\dagger }X}$ is the trace norm of the operator *X*. Negativity ranges from 0 to 1, where 1 is the negativity of a maximally entangled states and 0 is the Negativity of a completely separable states. For the family of states taken into account (Eq. 1), the Negativity becomes:

3 $${\mathscr{N}}=2p\sqrt{q(1-q)}.$$

Exploiting the Quantum Fisher Information it is possible calculate the quantum Cramér-Rao bound for the estimation of the Negativity:

4 $$QCR{B}_{\varepsilon {\mathscr{N}}}=1-{{\mathscr{N}}}^{2},$$

representing the minimum variance obtained for the estimation of Negativity in a single measurement. Thus, the optimal estimation of Negativity presents as associated uncertainty:

5 $$un{c}_{Opt\varepsilon {\mathscr{N}}}=\pm \frac{\sqrt{QCR{B}_{\varepsilon {\mathscr{N}}}}}{\sqrt{n}}=\pm \frac{\sqrt{1-{{\mathscr{N}}}^{2}}}{\sqrt{n}}$$

where *n* represent the number of measurements.

We define a non-optimal estimator $\varepsilon {{\mathscr{N}}}_{1}$:

6 $$\varepsilon {{\mathscr{N}}}_{1}=1-4P(++),$$

where *P*(*x*) is the probability of the event *X* and, from now on, the symbol +(−) indicates projection onto the state $|+(-)\rangle =\frac{|H\rangle +(-)|V\rangle }{\sqrt{2}}$. In practice, the probability to find a pair of photons both with diagonal polarisation is calculated as: $P(++)=Nc(++)/(Nc(++)+Nc(+-)+Nc(-+)+Nc(--))$. Here *Nc* indicates the number of detected photon pairs (number of coincidences). In order to determine such probabilities, a large number of measurements on identical copies of the quantum state is needed.

The theoretical minimum uncertainty associated to the non-optimal estimator $\varepsilon {{\mathscr{N}}}_{1}$ is:

7 $$un{c}_{\varepsilon {{\mathscr{N}}}_{1}}=\pm \frac{\sqrt{-({{\mathscr{N}}}^{2}+2{\mathscr{N}}-3)}}{\sqrt{n}}.$$

Then, we define an optimal estimator $\varepsilon {{\mathscr{N}}}_{2}$:

8 $$\varepsilon {{\mathscr{N}}}_{2}=P(+-)+P(-+)-P(++)-P(--),$$

whose theoretical minimum uncertainty corresponds to the one set by the saturation of the quantum Cramér-Rao bound (Eq. 5).

## Log-Negativity

This parameter is defined as:

9 $$ {\mathcal L} =lo{g}_{2}(\Vert {\rho }^{{T}_{A}}\Vert ).$$

For the family of states taken into account, the Log-Negativity can be expressed as:

10 $$ {\mathcal L} =lo{g}_{2}\mathrm{(2}p\sqrt{q\mathrm{(1}-q)}+\mathrm{1)}.$$

The corresponding quantum Cramér-Rao bound is:

11 $$QCR{B}_{\varepsilon  {\mathcal L} }=-\frac{{2}^{- {\mathcal L} }({2}^{ {\mathcal L} }-2)}{{\mathrm{log}}^{2}\mathrm{(2)}}$$

We define the non-optimal estimator $\varepsilon { {\mathcal L} }_{1}$:

12 $$\varepsilon { {\mathcal L} }_{1}={\mathrm{log}}_{2}(1-4(P(++)-\frac{1}{4}))$$

presenting the following minimum uncertainty:

13 $$un{c}_{\varepsilon { {\mathcal L} }_{1}}=\pm \sqrt{-\frac{{4}^{- {\mathcal L} }({4}^{ {\mathcal L} }-4)}{{\mathrm{log}}^{2}\mathrm{(2)}}}\frac{1}{\sqrt{n}}$$

Moreover, we define the optimal estimator $\varepsilon { {\mathcal L} }_{2}$:

14 $$\varepsilon { {\mathcal L} }_{2}={\mathrm{log}}_{2}\,(1-(P(--)-P(-+)-P(+-)+P(++))).$$

The theoretical uncertainty associated to this estimator corresponds to the square root of the quantum Cramér-Rao bound (Eq. 11) for the Log-Negativity:

15 $$un{c}_{\varepsilon { {\mathcal L} }_{2}}=\pm \sqrt{-\frac{{2}^{- {\mathcal L} }({2}^{ {\mathcal L} }-2)}{{\mathrm{log}}^{2}\mathrm{(2)}}}\frac{1}{\sqrt{n}}.$$

## Concurrence

Concurrence is defined as:

16 $${\mathscr{C}}=max\mathrm{(0,}\,{\lambda }_{1}-{\lambda }_{2}-{\lambda }_{3}-{\lambda }_{4}),$$

where *λ*_*i*_ are eigenvalues of the matrix $R=\sqrt{\sqrt{\rho }({\sigma }_{y}\otimes {\sigma }_{y}){\rho }^{\ast }({\sigma }_{y}\otimes {\sigma }_{y})\sqrt{\rho }}$ in descending order, and *σ*_*y*_ is the Pauli matrix $$(\begin{array}{cc}0 & i\\ -i & 0\end{array})$$. For the family of states described by Eq. 1, the Concurrence becomes:

17 $${\mathscr{C}}=2p\sqrt{q\mathrm{(1}-q)}.$$

It is interesting to note that, for the family of states taken into account, Concurrence and Negativity have the same theoretical values. This non trivial result leads to the same quantum Cramér-Rao bound associated with Negativity (Eq. 4). Since both Negativity and Concurrence are described by Eq. (17), we can exploit the same estimators previously introduced in Eqs 6 and 8 even for the quantification of the Concurrence.

## Quantum Geometric Discord

As previously stated, we are also interested in the amount of discord of a state. In order to present a valid estimation technique for all the bipartite states represented by Eq. 1, we use the Quantum Geometric Discord (${\mathscr{Q}}$). This geometrical approximation is the best indicator for the discord amount in the states under test, and can be expressed in the following form:

18 $${\mathscr{Q}}=2{p}^{2}\mathrm{(1}-q)q$$

In analogy with the parameters quantifying the amount of entanglement, one could note that the Quantum Geometric Discord can be written as a function of Negativity:

19 $${\mathscr{Q}}=\frac{{{\mathscr{N}}}^{2}}{2}.$$

This result, true for the family of states in Eq. 1 but not in general, immediately allows calculating the corresponding quantum Cramér-Rao bound:

20 $$QCR{B}_{\varepsilon {\mathscr{Q}}}=\mathrm{2(1}-2{\mathscr{Q}}){\mathscr{Q}},$$

as well as the non-optimal estimator $\varepsilon {{\mathscr{Q}}}_{1}$ and the optimal estimator $\varepsilon {{\mathscr{Q}}}_{2}$:

21 $$\varepsilon {{\mathscr{Q}}}_{1}=\frac{1}{2}{(1-4P(++))}^{2},\,\,\,\,\varepsilon {{\mathscr{Q}}}_{2}=\frac{1}{2}{(P(+-)+P(-+)-P(++)-P(--))}^{2}.$$

The theoretical uncertainty associated to the non-optimal estimator $\varepsilon {{\mathscr{Q}}}_{1}$ is:

22 $$un{c}_{\varepsilon {{\mathscr{Q}}}_{1}}=\pm \sqrt{-2{\mathscr{Q}}(2{\mathscr{Q}}+2\sqrt{2}\sqrt{{\mathscr{Q}}}-3)}\frac{1}{\sqrt{n}}.$$

The theoretical uncertainty associated to the optimal estimator $\varepsilon {{\mathscr{Q}}}_{2}$ is the one saturating the quantum Cramér-Rao bound (Eq. 20):

23 $$un{c}_{\varepsilon {{\mathscr{Q}}}_{2}}=\pm \sqrt{\mathrm{2(1}-2{\mathscr{Q}}){\mathscr{Q}}}\frac{1}{\sqrt{n}}.$$

## Experimental Apparatus

The family of entangled states investigated in our work is constituted by two-photon polarization-entangled states obtained exploiting the phenomenon of spontaneous parametric down conversion (SPDC).

**Figure 1:**
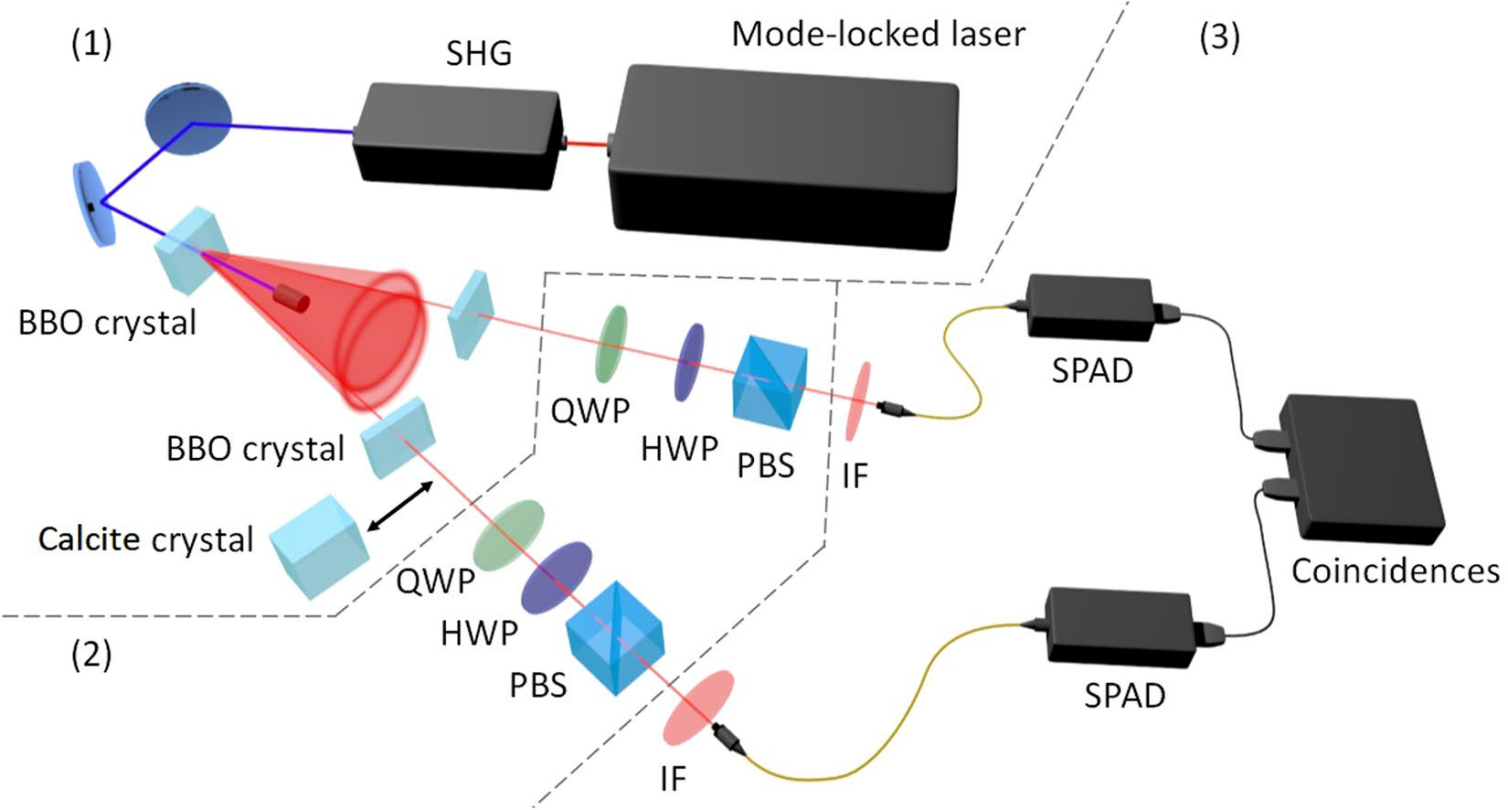
The experimental apparatus can be divided in three parts. (1) A source of polarisation entangled photons, based on SPDC, composed by: a Ti:sapphire mode-locked laser, a second harmonic generator (SHG), a BBO non-linear crystal for SPDC generation and two BBO crystals for walk-off compensation. A 2.7 mm length birefringent crystal, with optical axis orthogonal to the photon propagation direction, can be inserted in one of the paths in order to introduce decoherence. (2) A tomographic measurement apparatus, where each branch hosts: a quarter wave plate (QWP), a half wave plate (HWP) and a polarising beam splitter (PBS). (3) The detection system, comprising two interference filters (IF), two fiber couplers injecting the photons into multi-mode fibers addressing them to Silicon single-photon avalanche diodes (SPADs), and coincidence electronics.

The first part of the set-up (corresponding to the region (1) in Fig. 1) is a source of polarization-entangled photons based on a scheme^53^ exploited in many experiments concerning foundations of quantum mechanics and quantum technologies^24^. In particular, our scheme is based on a Ti:Sapphire mode-locked laser, emitting pulses with duration of 150 fs at a wavelength centred on 808 nm. Such laser beam induces the second harmonic generation in a lithium triborate (LBO) non-linear crystal. The resulting beam, with a central wavelength at 404 nm, is used to pump a 0.5 mm long *β*-barium borate (BBO) non-linear crystal where type-II SPDC occurs, generating correlated photon pairs^54^. Two irises are used to spatially select the photons belonging to the intersections of the horizontally- and vertically-polarized degenerate SPDC cones (808 nm). On each of the two selected paths, a 0.25 mm thick BBO crystal is used to compensate the temporal delay between the horizontally- and the vertically-polarized photons induced by the birefringence within the SPDC crystal. At the output of these crystals, ideally, the polarisation-entangled photons are in the state:

24 $$|{\psi }_{\varphi }\rangle =\frac{|HV\rangle +{e}^{i\varphi }|VH\rangle }{\sqrt{2}}$$

(being H and V, respectively, the horizontal and vertical polarisation components), with a relative phase *ϕ* between the ordinary and extraordinary polarized light. A fine tilting of one of the compensation crystals is performed to tune the parameter *ϕ*.

It is possible to introduce decoherence in our entangled state by introducing, in one of the two paths, an additional birefringent crystal sufficiently thick (for this purpose we use a 2.7 mm thick calcite crystal).

The second part of the setup (corresponding to the region (2) in Fig. 1) is a typical polarisation quantum tomographic apparatus^55^. Each path is equipped with a quarter wave plate (QWP), a half wave plate (HWP) and a polarising beam splitter (PBS), allowing to project each photon polarisation onto any state of the Bloch sphere surface.

Finally (in the region (3) of Fig. 1), for each path, an interference filter (IF) spectrally selects the photons, subsequently injected into a multi-mode fibre. Then, the fibre sends the photons to a Silicon single-photon avalanche diode (SPAD) for the detection. A dedicated time correlated counting system is used to perform temporal post-selection on photon counts.

## Results

In our experiment, we do not physically produce the set of quantum states described in Eq. 1. Experimentally, we realise (independently and at different times) pure singlet states $|{\psi }_{-}\rangle$ and completely decoherent states *ρ*_*mix*_. To explore states with different amount of decoherence, we realise a statistical mixture of our data in post-processing. Therefore, for both physical pure singlet state $|{\psi }_{-}\rangle$ and completely decoherent state, we perform all the measurements required by the estimators we are interested in, with an appropriate redundancy. Then, in post-processing, we mix the results in different percentage in order to simulate the right quantum mixture (a technique already exploited in several experiments^42,56^, proven to give results indistinguishable from the ones obtained measuring a physical mixed state).

To determine the quality of the states produced in our experiment, we exploit the quantum state tomography technique and we calculate the Uhlmann’s Fidelity^57^ of the reconstructed state with respect to the theoretical expectations:

25 $$ {\mathcal F} =Tr(\sqrt{\sqrt{{\rho }^{exp}}{\rho }^{th}\sqrt{{\rho }^{exp}}}).$$

**Figure 2:**
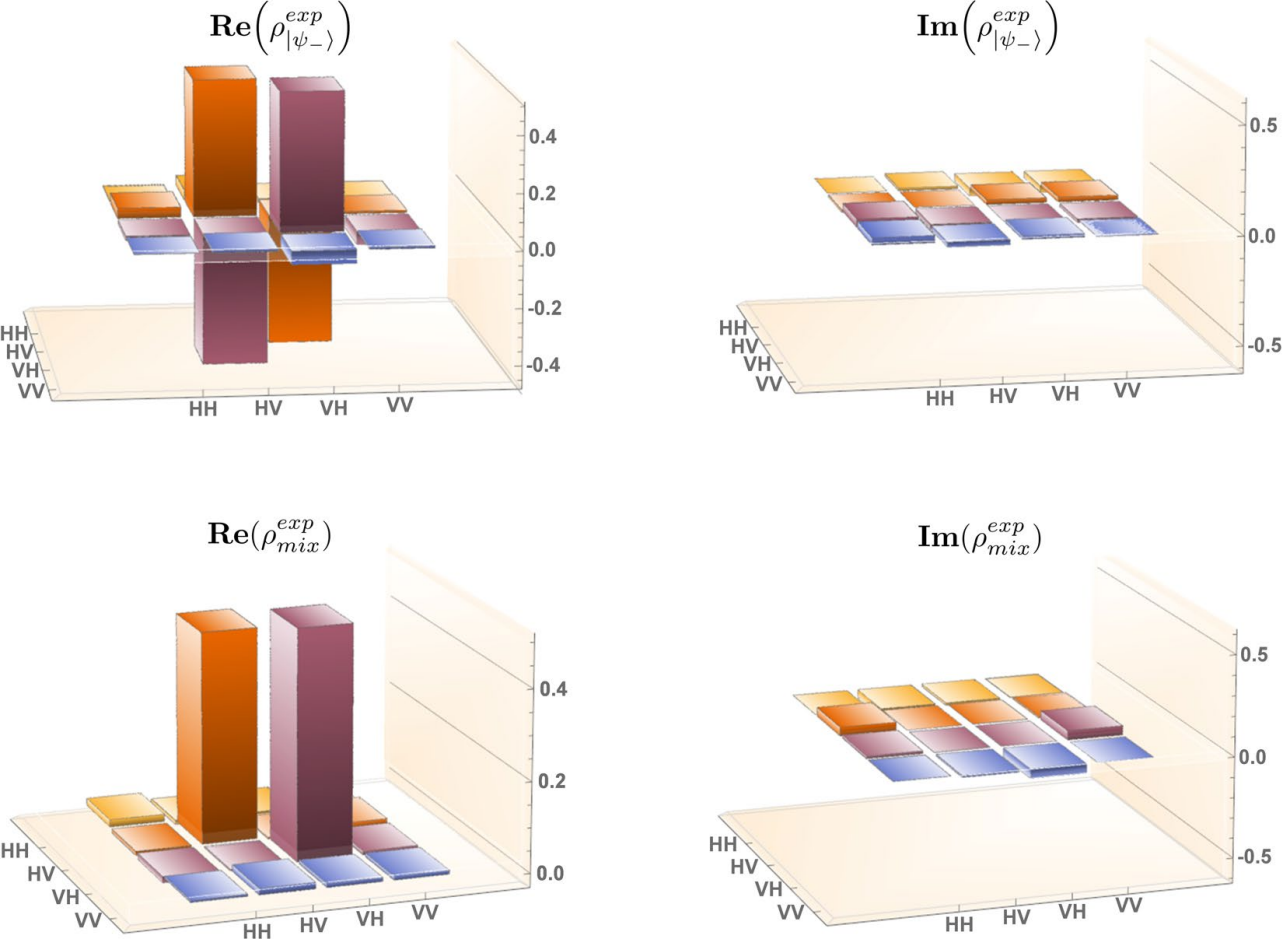
Real (left) and imaginary (right) part of the tomographically reconstructed density matrix for the singlet, maximally-entangled state (top) and the completely decoherent mixture (bottom).

Here, *ρ*^*exp*^ is the reconstructed density matrix and *ρ*^*th*^ is the corresponding theoretical one. The experimentally reconstructed matrices of the singlet state and of the decoherent mixture generated in our setup are shown in Fig. 2, while the corresponding theoretical matrices can be written respectively, in the H-V basis, as:

26 $$\begin{array}{ccccc}{\rho }_{|{\psi }_{-}\rangle }^{th} & = & (\begin{array}{cccc}0 & 0 & 0 & 0\\ 0 & 1/2 & -1/2 & 0\\ 0 & -1/2 & 1/2 & 0\\ 0 & 0 & 0 & 0\end{array}) & \,\,\,\, & {\rho }_{mix}^{th}=(\begin{array}{cccc}0 & 0 & 0 & 0\\ 0 & 1/2 & 0 & 0\\ 0 & 0 & 1/2 & 0\\ 0 & 0 & 0 & 0\end{array}),\end{array}$$

where the choice to operate with a singlet states implies *q* = 1/2 (see Eq. 1).

The Fidelity values obtained are ${ {\mathcal F} }_{{\psi }_{-}}=0.975$ and ${ {\mathcal F} }_{{\psi }_{mix}}=0.985$ respectively.

**Figure 3:**
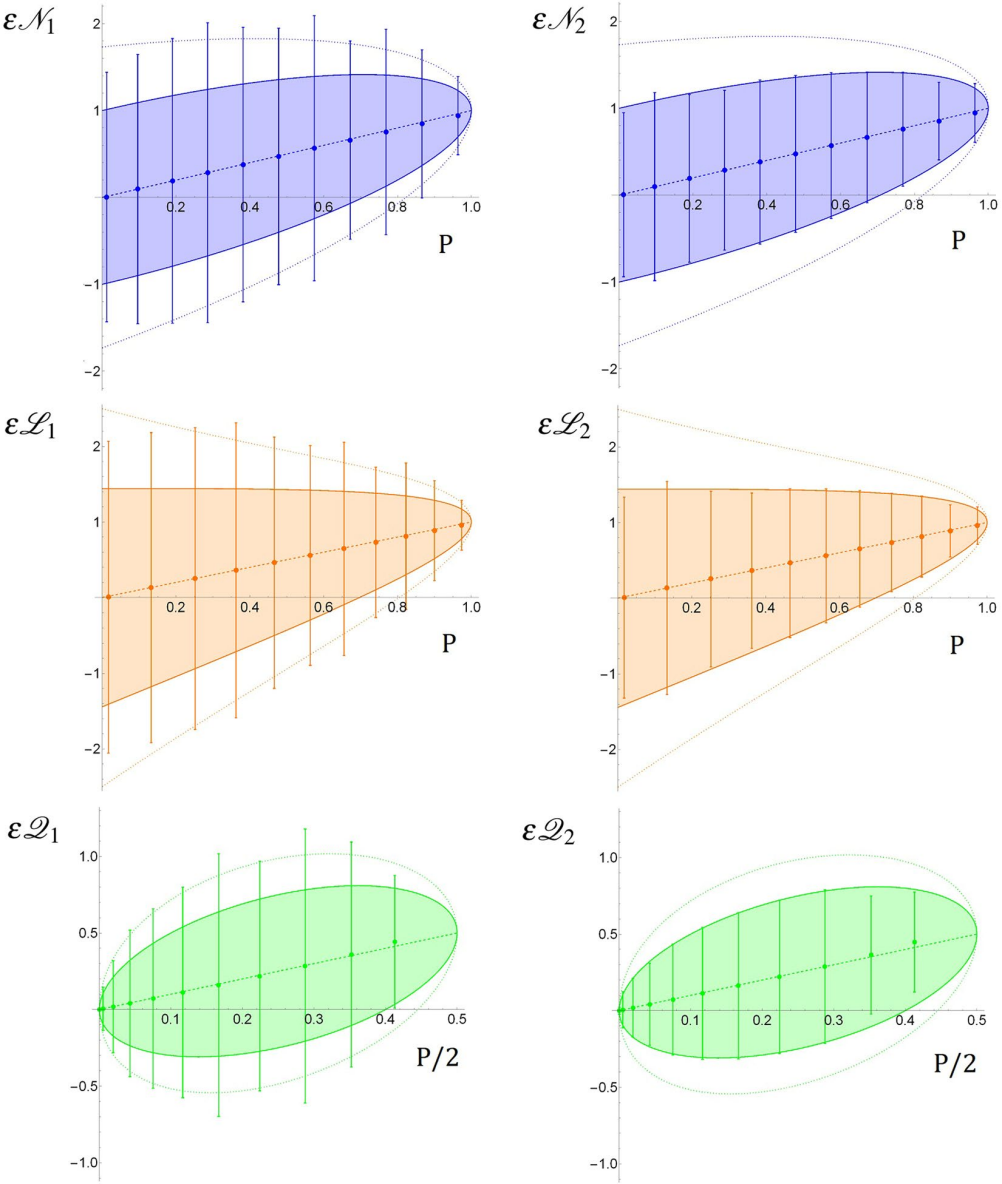
Results for Negativity and Concurrence (blue), Log-Negativity (red), and Quantum Geometric Discord (green) non-optimal (left side) and optimal (right side) estimators, with respect to *p* (see Eq. 1). Experimental points are compared with: theoretical value of the quantity to estimate (dashed line), theoretical uncertainty for the non-optimal estimator (dotted curve) and theoretical uncertainty related to the quantum Cramér-Rao bound (solid curve).

In Fig. 3 are shown the main experimental results of this work. The experimental points concerning the several estimators introduced in this paper are plotted in function of the mixing parameter *p* (defined in Eq. 1) ranging from 0 (completely decoherent mixture) to 1 (pure entangled state). For each point, the value of *p* is evaluated exploiting the tomographical reconstruction of the density matrix of the corresponding quantum state. Each point results from the average on 10 independent estimations. The uncertainty bars associated with the experimental points represent the standard deviation of the measurement results statistical distribution, i.e. the statistical uncertainty associated with a single measurement. Experimental points are compared with the theoretical value of the estimator, represented by a dashed line. The experimental uncertainty bars are compared with the theoretical value of the uncertainty derived by the quantum Fisher information. Dotted curves represent the theoretical uncertainty for the non-optimal estimator, while solid curves indicate the theoretical uncertainty for the optimal estimator, i.e. the one saturating the quantum Cramér-Rao bound, representing the minimum uncertainty allowed by quantum estimation theory. All the theoretical curves shown in Fig. 3 are calculated exploiting the knowledge of the experimental values of the parameters *p* and *q*, obtained from the tomographical reconstruction of the density matrices (see Fig. 2) of the physical systems involved in the experiment.

In Fig. 3 different colours have been used for different parameters, in particular: blue for Negativity and Concurrence, orange for Log-Negativity and green for Quantum Geometric Discord. On the left side of Fig. 3 are shown the plots concerning the non-optimal estimators for each parameter, while on the right side are shown the optimal estimators plots. The figure shows a good agreement between experimental results and theoretical predictions for each estimator, both for the value itself and the statistical uncertainty associated with it. This is particularly relevant and interesting for the optimal estimators case, where our results demonstrate saturation of the Quantum Cramér-Rao bound.

## Conclusion

We performed an experiment comparing several non-classicality parameters related either to entanglement or discord. We directly extract the amount of entanglement with Negativity, Concurrence and Log-Negativity, while we approximately evaluate the amount of discord by estimating the Quantum Geometric Discord. For each of these quantities we introduce two estimators, a non-optimal one and an optimal one, for a particular family of states that have a recognised importance in the field of quantum information and related technologies. By evaluating the statistical uncertainties as the standard deviations on repeated measurements, we achieve a good agreement between the theoretical predictions and the experimental results. In particular, we demonstrate that optimal estimators reach the ultimate theoretical precision limit represented by the quantum Cramér-Rao bound. The agreement between uncertainty bars and theoretical uncertainty curves, also for what concerns non-optimal estimators, represents a further check on the consistency between our experimental data and the theory.

It is possible to note a significant reduction of the uncertainties between the optimal and the non-optimal estimators, demonstrating a strong and practical advantage in the use of the optimal estimators. These results pave the way to the diffuse use of these estimators in quantifying resources for quantum technologies. A further remarkable result emerging from this work is that, for the family of quantum states taken into account, there is an identity between the estimators for Negativity and Concurrence, and a smooth monotone relation between the ones for Negativity and Quantum Geometric Discord. Such result was not expected a priori but leads to direct relations between estimators and related quantum Cramér-Rao bounds. Therefore, knowing the quantum Cramér-Rao bound and the optimal estimator for Negativity allows an immediate derivation of such quantities also for Log-Negativity, Concurrence and Quantum Geometric Discord.

## References

[1] Paris, M. G. A. & Rehacek, J. *Lecture Notes Physics*, vol. 649 (Springer, Berlin, 2004).

[2] Vidal, G. & Werner, R. F. *Computable measure of entanglement*, vol. 65 (2002).

[3] D’Ariano GM, Macchiavello C, Paris MGA (1994). Precision of quantum tomographic detection of radiation. Phys. Lett. A.

[4] Asorey M (2011). Robustness of raw quantum tomography. Phys. Lett. A.

[5] Horodecki R, Horodecki P, Horodecki M, Horodecki K (2009). Quantum entanglement. Rev. Mod. Phys..

[6] Gühne O, Toth G (2009). Entanglement detection. Phys. Rep..

[7] Augusiak R, Lewenstein M (2009). Towards measurable bounds on entanglement measures. Quant. Info. Proc..

[8] Horodecki P (2003). Measuring quantum entanglement without prior state reconstruction. Phys. Rev. Lett..

[9] Vedral V, Plenio MB, Rippin MA, Knight PL (1997). Quantifying entanglement. Phys. Rev. Lett..

[10] Wunderlich H, Plenio M (2009). Quantitative verification of entanglement and fidelities from incomplete measurement data. J. Mod. Opt..

[11] Eisert J, Brandao FGSL, Adenauert K (2007). Quantitative entanglement witnesses. New J. Phys.

[12] Audenaert K, Plenio MB (2006). When are correlations quantum?-verification and quantification of entanglement by simple measurements. New J. Phys.

[13] Lougovski P, van Enk S (2009). Characterizing entanglement source. Phys. Rev. A.

[14] Jaeger G, Horne M, Shimony A (1993). Complementarity of one-particle and two-particle interference. Phys. Rev. A.

[15] Clauser JF, Horne MA, Shimony A, Holt RA (1969). Proposed experiment to test local hidden-variable theories. Phys. Rev. Lett..

[16] Horodecki M, Horodecki P, Horodecki R (1996). Separability of mixed states: necessary and sufficient conditions. Phys. Lett. A.

[17] Terhal B (2000). Bell inequalities and the separability criterion. Physics Letters A.

[18] Gühne O (2002). Detection of entanglement with few local measurements. Phys. Rev. A.

[19] Brandao, F. G. S. L. & Vianna, R. O. Witnessed entanglement. *Int. Journ. Quant. Inf*. **331** (2006).

[20] Krammer P (2009). Multipartite entanglement detection via structure factors. Phys. Rev. Lett..

[21] Facchi P, Florio G, Pascazio S (2007). Characterizing and measuring multipartite entanglement. Int. J. Quantum. Inform..

[22] Fedorov M, Efremov M, Volkov P, Eberly J (2006). Short-pulse or strong-field breakup processes: a route to study entangled wave packets. J. Phys. B: At., Mol. Opt. Phys..

[23] Volkov PA, Mikhailova YM, Fedorov MV (2009). Spectral entanglement in parametric down-conversion with nondegenerate frequencies. Adv. Sci. Lett..

[24] Genovese M (2005). Research on hidden variable theories: A review of recent progresses. Phys. Reports.

[25] Bourennane M (2004). Experimental detection of multipartite entanglement using witness operators. Phys. Rev. Lett..

[26] Fedorov MV (2007). Anisotropically and high entanglement of biphoton states generated in spontaneous parametric down-conversion. Phys. Rev. Lett..

[27] Fedorov MV (2008). Spontaneous parametric down-conversion: Anisotropical and anomalously strong narrowing of biphoton momentum correlation distributions. Phys. Rev. A.

[28] Brida G (2009). Characterization of spectral entanglement of spontaneous parametric-down conversion biphotons in femtosecond pulsed regime. Europhys. Lett..

[29] Avenhaus M, Chekhova MV, Krivitsky LA, Leuchs G, Silberhorn C (2009). Experimental verification of high spectral entanglement for pulsed waveguided spontaneous parametric down-conversion. Phys. Rev. A.

[30] Barbieri M (2003). Detection of entanglement with polarized photons: Experimental realization of an entanglement witness. Phys. Rev. Lett..

[31] Walborn SP, Souto Ribeiro PH, Davidovich L, Mintert F, Buchleitner A (2006). Experimental determination of entanglement with a single measurement. Nat..

[32] Almeida MP (2007). Environment-induced sudden death of entanglement. Sci..

[33] Ollivier H, Zurek WH (2001). Quantum discord: A measure of the quantumness of correlations. Phys. Rev. Lett..

[34] Luo S (2008). Quantum discord for two-qubit systems. Phys. Rev. A.

[35] Jian-Song Z, Ai-Xi C (2012). Review of quantum discord in bipartite and multipartite systems. Quant. Phys. Lett..

[36] Giorda P, Paris MGA (2010). Gaussian quantum discord. Phys. Rev. Lett..

[37] Datta A, Shaji A, Caves CM (2008). Quantum discord and the power of one qubit. Phys. Rev. Lett..

[38] Benedetti C, Shurupov AP, Paris MGA, Brida G, Genovese M (2013). Experimental estimation of quantum discord for a polarization qubit and the use of fidelity to assess quantum correlations. Phys. Rev. A.

[39] Dakic, B. *et al*. Quantum discord as resource for remote state preparation. *Nat. Phys*. **8**, 666 EP – Article (2012).

[40] Girolami D, Adesso G (2011). Quantum discord for general two-qubit states: Analytical progress. Phys. Rev. A.

[41] Brida G (2011). Optimal estimation of entanglement in optical qubit systems. Phys. Rev. A.

[42] Brida G (2010). Experimental estimation of entanglement at the quantum limit. Phys. Rev. Lett..

[43] Paris MGA (2009). Quantum estimation for quantum technology. Int. J. Quantum Inf..

[44] Brody DC, Hughston LP (1999). Geometrization of statistical mechanics. Proc. Royal Soc. Lond. A: Math. Phys. Eng. Sci..

[45] Brody DC, Hughston LP (1998). Statistical geometry in quantum mechanics. Proc. Royal Soc. Lond. A: Math. Phys. Eng. Sci..

[46] Braunstein S, Caves C, Milburn G (1996). Generalized uncertainty relations: Theory, examples, and lorentz invariance. Annals Phys..

[47] Braunstein SL, Caves CM (1994). Statistical distance and the geometry of quantum states. Phys. Rev. Lett..

[48] Helstrom C (1967). Minimum mean-squared error of estimates in quantum statistics. Phys. Lett. A.

[49] Genoni MG, Giorda P, Paris MGA (2008). Optimal estimation of entanglement. Phys. Rev. A.

[50] Verstraete F, Audenaert K, Dehaene J, De Moor B (2001). A comparison of the entanglement measures negativity and concurrence. J. Phys. A: Math. Gen..

[51] Luo S, Fu S (2010). Geometric measure of quantum discord. Phys. Rev. A.

[52] Dakić B, Vedral V, Brukner ICV (2010). Necessary and sufficient condition for nonzero quantum discord. Phys. Rev. Lett..

[53] Kwiat PG (1995). New high-intensity source of polarization-entangled photon pairs. Phys. Rev. Lett..

[54] Boeuf, N. *et al*. Calculating characteristics of noncollinear phase matching in uniaxial and biaxial crystals. *Opt. Eng*. **39** (2000).

[55] Bogdanov YI (2011). Statistical estimation of the quality of quantum-tomography protocols. Phys. Rev. A.

[56] Carvacho G (2017). Experimental investigation on the geometry of GHZ states. Sci. Reports.

[57] Jozsa R (1994). Fidelity for mixed quantum states. J. Mod. Opt..

